# Drugs developed to treat diabetes, liraglutide and lixisenatide, cross the blood brain barrier and enhance neurogenesis

**DOI:** 10.1186/1471-2202-13-33

**Published:** 2012-03-23

**Authors:** Kerry Hunter, Christian Hölscher

**Affiliations:** 1 School of Biomedical Sciences, Ulster University, Coleraine, UK; 2 University of Ulster, School of Biomedical Sciences, Cromore Road, Coleraine BT52 1SA, Northern Ireland

**Keywords:** Alzheimer disease, Parkinson's disease, Diabetes, Neuroprotection, Stem cells

## Abstract

**Background:**

Type 2 diabetes is a risk factor for Alzheimer's disease (AD), most likely linked to an impairment of insulin signalling in the brain. Therefore, drugs that enhance insulin signalling may have therapeutic potential for AD. Liraglutide (Victoza) and exenatide (Byetta) are novel long-lasting analogues of the GLP-1 incretin hormone and are currently available to treat diabetes. They facilitate insulin signalling via the GLP-1 receptor (GLP-1R). Numerous *in vitro *and *in vivo *studies have shown that GLP-1 analogues have a range of neuroprotective properties. GLP-1Rs are expressed in the hippocampal area of the brain an important site of adult neurogenesis and maintenance of cognition and memory formation. Therefore, if GLP-1 analogues can cross the blood brain barrier, diffuse through the brain to reach the receptors and most importantly activate them, their neuroprotective effects may be realized.

**Results:**

In the present study we profiled the GLP-1 receptor agonists liraglutide (Victoza) and lixisenatide (Lyxumia). We measured the kinetics of crossing the blood brain barrier (BBB), activation of the GLP-1R by measuring cAMP levels, and physiological effects in the brain on neuronal stem cell proliferation and neurogenesis. Both drugs were able to cross the BBB. Lixisenatide crossed the BBB at all doses tested (2.5, 25, or 250 nmol/kg bw ip.) when measured 30 min post-injection and at 2.5-25 nmol/kg bw ip. 3 h post-injection. Lixisenatide also enhanced neurogenesis in the brain. Liraglutide crossed the BBB at 25 and 250 nmol/kg ip. but no increase was detectable at 2.5 nmol/kg ip. 30 min post-injection, and at 250 nmol/kg ip. at 3 h post-injection. Liraglutide and lixisenatide enhanced cAMP levels in the brain, with lixisenatide being more effective.

**Conclusions:**

Our results suggest that these novel incretin analogues cross the BBB and show physiological activity and neurogenesis in the brain, which may be of use as a treatment of neurodegenerative diseases.

## Background

Type 2 diabetes mellitus (T2DM) is characterized by insulin resistance resulting in glucose intolerance and hyperglycaemia [1].

Since insulin effectiveness is reduced in diabetes, research into other signalling pathways that support insulin actions or that reduce blood glucose is ongoing. One of these strategies focus on the use of the incretins, a class of peptide hormones that helps to normalise insulin signaling and also improves blood sugar levels. Incretins increase the release of insulin during high blood sugar levels, the so-called 'incretin effect'. Drugs that mimic incretin hormones can maintain glucose homeostasis and improve multiple symptoms of type 2 diabetes like the risk of hypoglycaemia, inadequate post-prandial blood glucose control, glucose fluctuations, β-cell failure, and weight gain [1,2].

GLP-1 is an endogenous 30-amino acid peptide hormone. Numerous novel long-lasting GLP-1 receptor agonists have been developed by several companies. Exendin-4 (Byetta) has been on the market as a T2DM treatment for several years [3]. Liraglutide (Victoza) also has been released onto the market several years ago [4]. A third drug is lixisenatide (Lyxumia), which will be released onto the market soon [5].

T2DM has been identified as a risk factor for AD, indicating that insulin signaling failure may be a factor in initiating or accelerating the development of AD. Epidemiological studies found a clear correlation between T2DM and the risk of developing AD [6-8]. It was also shown that insulin receptors in the brain are desensitised in AD patients [9,10]. Therefore, a promising strategy to treat AD is the use of such GLP-1 analogues [11]. GLP-1 receptors are found on neurons in the brains of rodents and humans [12,13]. The GLP-1 receptor agonists exendin-4, liraglutide, lixisenatide, and (Val8)GLP-1 have neuroprotective properties. The protease resistant and long-lasting GLP-1 analogue Val(8)GLP-1 enhanced synaptic plasticity in acute and chronic application and preserved synaptic functionality in the brains of a mouse model of AD [14,15]. The novel GLP-1 analogue liraglutide lowered plaque formation, protected memory and synaptic plasticity, and reduced inflammation in the brains of a mouse model of Alzheimer disease [16]. All of these effects in the brain were observed after peripheral injection. Therefore, it is likely that these peptides have to be able to cross the blood brain barrier (BBB).

The focus of this study was to measure the kinetics of incretin drugs of crossing the blood brain barrier, activation of incretin receptors by measuring cAMP levels, and physiological effects in the brain on cell proliferation and neurogenesis.

## Results

### Experiment 1

**Figure 1:**
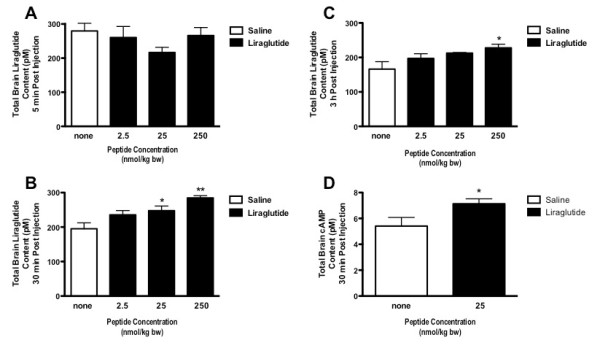
**At 5 min post- ip. injection, there was no significant increase of liraglutide in the brains of mice (A)**. There was an increase in liraglutide levels in the brains of mice injected with 25 or 250 nmol/kg bw at 30 min **(B) **and only those injected with the highest dose at 3 h post-injection **(C)**. Brain levels of cAMP were increased 30 min post injection with 25 nmol/kg bw ip.. * = *p *< 0.05. Values are the mean ± S.E.M.

There were no significant levels of Liraglutide found 5 min post i.p. injection, in the brains of mice administered with a 2.5 nmol/kg bw, 25 nmol/kg bw or 250 nmol/kg bw dose of the peptide (see Figure 1a). However, significant levels of the GLP-1R agonist were found at both 30 min and 3 h post injection with the 250 nmol/kg dose (*p *< 0.01 and *p *< 0.05 respectively). The medium dose of 25 nmol/kg bw was also sufficient to raise peptide levels in the brain 30 min post injection (*p *< 0.05) but not after 3 h (Figure 1b and 1c). To determine whether Liraglutide activates the GLP-1R in the brain we measured total brain cAMP levels 30 min post i.p. injection with 25 nmol/kg bw of the peptide. cAMP is the second messenger of the GLP-1R. Significant levels of cAMP (*p *< 0.05) were detected 30 min post administration indicating GLP-1R activation (Figure 1d).

### Experiment 2

**Figure 2:**
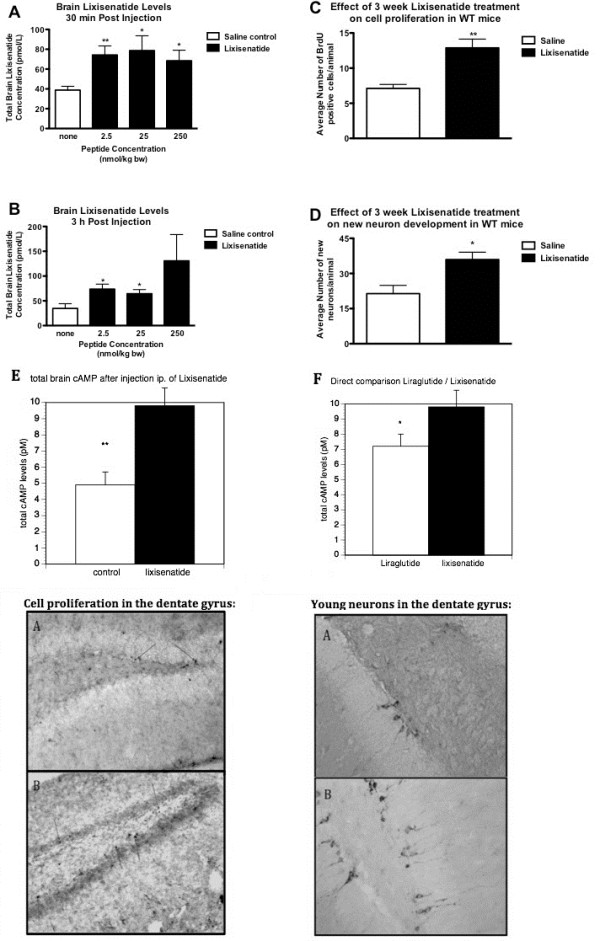
**(A) Lixisenatide levels in the brains at 30 min following i.p. (2.5, 25 or 250 nmol/kg body weight) injection were increased**. **(B) **Lixisenatide levels in the brains at 3 h following i.p. injection were only increased for the lower doses (2.5, 25 nmol/kg body weight). **(C) **The number of neuronal progenitor cells in the dentate gyrus was increased after 3 weeks of ip injection 25 nmol/kg bw once-daily. **(D) **The number of young neurons in the dentate gyrus was also increased. **(E) **The level of cAMP was enhanced in the brains after injection with 25 nmol/kg bw ip. **(F) **When directly comparing the effects of liraglutide with lixisenatide, a significant difference between drugs is found.* = *p *< 0.05, ** = *p *< 0.01. **Micrographs: **BrdU stain: A Saline control B Lixisenatide treated, 3 weeks once daily i.p. injection. Blue arrows point to BrdU positively stained cells. DCX stain: Doublecortin stained immature neurons in dentate gyrus. A Saline control B Lixisenatide treated, 3 weeks once daily i.p. administration.

Lixisenatide was found in significant concentrations following administration with 2.5, 25 or 250 nmol/kg bw of the peptide at 30 min post injection (*p *< 0.01, *p *< 0.05 and *p *< 0.05) (Figure 2a). Similarly at 3 h post administration Lixisenatide was still present in a significant amount in the brain 3 h post injection in the mice given the 2 lower doses (*p *< 0.05), however, not with the highest dose due to huge variation within this particular group (Figure 2b). Interestingly, for this peptide there is as much detected in the brain when administered the lowest dose of 2.5 nmol/kg bw as with the highest dose of 250 nmol/kg bw. Furthermore, there is little or no decrease in the concentration of Lixisenatide from 30 min to 3 h post injection.

There was a 1.8-fold increase in cell proliferation as measured by BrdU analysis (*p *< 0.01, Figure 2c). Correspondingly, there was a 1.7-fold increase (*p *< 0.05) in young neurons as determined by Double Cortin analysis (Figure 2d). The level of cAMP was inhanced in the brains after injection with 25 nmol/kg bw ip. (*p *< 0.01), see Figure 2e. When directly comparing the effects of liraglutide with lixisenatide, a significant difference between drugs is found (*p *< 0.05, Figure 2f).

## Discussion

We have investigated the GLP-1 receptor agonists Liraglutide and Lixisenatide. We have provided clear evidence that both peptides have the ability to cross the BBB, as we found significantly elevated levels of these peptides in the brain 30 min following i.p. injection. Liraglutide levels in the brain were higher with increasing doses injected ip. Also, there was a time-dependence as only the highest dose injected showed a significant increase in the brain after 3 h. The brain levels of cAMP were increased, demonstrating the activation of the GLP-1 receptor which is linked to an adenylate cyclase [1]. Previous studies already had demonstrated that liraglutide also enhances neuronal progenitor proliferation and increased neurogenesis in the brain of a mouse model of Alzheimer disease [16].

Lixisenatide also entered the brain in a sufficiently detectable amount, even at the lowest dose tested of 2.5 nmol/kg bw. 30 min post-injection. At 3 h post-injection, the highest dose did not show a significant increase in the brain. This observation suggests that the transport across the BBB is highly regulated, and that this transport system may shut down at doses that are abnormally high. Similar observations have been made previously when testing exendin-4 [17]. The brain levels of cAMP were increased, demonstrating the activation of the GLP-1 receptor. We were also able to demonstrate that lixisenatide, when injected once daily for 3 weeks, promotes neuronal progenitor proliferation in the dentate gyrus, and neurogenesis in the brain, demonstrating that this drug not only crosses the BBB, but also shows clear physiological effects in the brain. Interestingly, lixisenatide is transported across the BBB at a lower dose than liraglutide, and shows enhanced cAMP at equal dose compared with liraglutide.

Our results show that incretin analogues can be injected peripherally and are taken up into the brain where they show physiological activity. Previous studies have shown similar effects in the brain, in particular on cell proliferation and neurogenesis [14,18-21], and our results add considerably to those findings.

Exendin-4 is currently on the market as a T2DM treatment and is injected peripherally twice-daily. Liraglutide is also on the market and is injected peripherally once daily [1]. Lixisenatide is under development to be used as a once-daily treatment for T2DM [5]. Such drugs can be taken by non-diabetic people as they only affect blood sugar levels in hyperglycaemia [22].

## Conclusion

If the improvements in memory and synaptic plasticity, reduction in neurotoxic amyloid oligomer levels, numbers of amyloid plaques and of the inflammation response, and increased neurogenesis observed in previous studies [11,23] translate to humans, GLP-1 analogues such as Liraglutide or GLP-1 receptor agonists such as exendin-4 and lixisenatide are promising new treatment strategies for Alzheimer's disease. Importantly, clinical trials of the effects of exendin-4 in patients with Parkinson's disease (UCL, London, UK) have been started, and clinical trials in patients with MCI or early-phase AD are on their way (NIH/NIA, USA) (see an update on http://www.clinicaltrials.gov). A third clinical trial that aims to test liraglutide in AD patients is currently being prepared.

## Methods

### Experiment 1

#### Animals

Each animal received saline by i.p. injection (0.9% (w,v) NaCl) or liraglutide (2.5 nmol/kg body weight (bw), 25 nmol/kg bw or 250 nmol/kg bw) (female, C57/BL 6 background, n = 5 per group). At 5 min, 30 min or 3 h post treatment the mice were painlessly killed with an overdose of anaesthetic. Subsequently, animals were perfused with PBS in order to clear the circulation of all blood. Each brain was weighed, placed in a Bijou tube, snap frozen in liquid nitrogen and stored at -80 C until further processing and analysis. All experiments were licenced by the UK home office (Project licence No. PPL2603b).

#### Acid ethanol extraction of liraglutide from mouse brains

Tubes containing the brains from the mice were removed from storage and kept in liquid nitrogen. Each tube was processed one at a time to avoid thawing. 2.5 ml of acid ethanol (75% ethanol, 1.5% conc HCl)/g of tissue was added and the sample sonicated using a Soniprep 150 plus HSE (Davidson and Harley Ltd). Samples were stored on ice before centrifugation at 10,000 rpm for 15 min at 4 C using the Beckman centrifuge. The supernatant was poured off into fresh eppendorf tubes. 300 μl of the supernatant was removed to a clean eppendorf tube and 300 μl of 1 M Tris (pH 7.6) was added. These samples were then dried down in a Speedvac at 45 C for 1 h 30 min to completely remove the liquid. Samples were reconstituted in GLP-1 ELISA kit assay buffer (Millipore, MA, USA).

#### ELISA analysis of brain samples

The GLP-1 (Active) 96 well fluorescent ELISA kit (EGLP-35 K, Millipore, MA, USA) was used to measure the Liraglutide levels detected in the brain samples. The assay was performed according to the manufacturers instructions. Briefly, the plate was incubated with wash buffer for 5 min then decanted and the excess removed by tapping on absorbent towels. Assay buffer was added to all wells followed by either the standards, quality controls or samples in the appropriate order on the plate. The plate was covered with a plate sealer, gently shaken then incubated overnight at 4^°^C. On day 2 the plate was washed 5 times with wash buffer, detection conjugate added and incubated at room temperature for 2 h. The plate was then washed 3 times and diluted substrate added and allowed to incubate in the dark for 18 min. Stop solution was added and the plate read at 355 nm/460 nm using the Flexstation 3 and Softmax programme. Brain samples were diluted 1:10 prior to measurement on the plate so results were adjusted accordingly following analysis.

#### cAMP analysis of brain samples (for both experiments)

Each brain was extracted using 0.1 M HCl. 10 ml of 0.1 M HCl per g of tissue was added. Samples were sonicated then centrifuged at 10,000 rpm for 15 min at 4^°^C. The supernatent was poured off and used directly for measurement by ELISA. Dilutions were made using the 0.1 M HCl provided in the kit. Brain cAMP levels were measured using the direct cAMP ELISA kit (Enzo Life Sciences) according to the manufacturers instructions.

### Experiment 2

We examined the concentration of Lixisenatide found in the brains of female WT mice following i.p. administration of 2.5, 25 or 250 nmol/kg bw of the peptide at 30 min and 3 h post injection. We also undertook a 3 week study on the effect of 25 nmol/kg bw daily administration of Lixisenatide on cell proliferation and new neuron development in the brains of WT mice.

#### Animals

In the chronic study, one group of female WT mice (n = 6) received once daily Lixisenatide (25 nmol/kg bw) for 3 weeks and the other group (n = 6) received saline (0.9% (w,v) NaCl) as control. At the end of the treatment period the mice were painlessly killed with an overdose of anaesthetic. Subsequently, animals were perfused with PBS, then with paraformaldehyde (PFA). Each brain was divided into 2 hemispheres, stored in PFA and kept at 4°C until further processing and analysis. In order to perform BrdU immunohistological analysis, animals were injected with 5-Bromo-2-DeoxyUridine (BrdU) (50 mg/kg) 24 h prior to sacrificing. Acid ethanol extraction of Lixisenatide from mice brains was performed as described above.

#### ELISA analysis of brain samples

Analysis of the Lixisenatide treated brains was carried out as recommended by the manufacturer of the total GLP-1 (7-36 and 9-36) ELISA kit, 43-GPTHU-E01, (Alpco. NH, USA). Briefly, standards, controls and test samples were added to the designated wells and then antibody mixture was added to each of these wells. The plate was sealed and incubated overnight at 4^°^C. Contents were then removed and the plate washed 5 times before adding HRP substrate. The plate was protected from exposure to light and incubated at RT for 20 min. Stop solution was then added, mixed gently and the absorbance read at 450 nm/620 nm within 10 min.

#### Cryostat preparation of brain tissue

One hemisphere from each animal was transferred from the PFA and placed in 30% sucrose solution overnight at 4^°^C. Each hemisphere was then mounted on the specimen disc using tissue-tek and Envi Ro Tech freeze spray. Brain sections were cut at 45 μm and placed in cryoprotect solution. Every sixth section was taken for analysis.

#### BrdU Immunohistochemical analysis

Cryoprotection solution was removed and sections washed in diH2O then incubated in H2O2. Sections were rinsed in H2O then incubated in 0.3% Triton X for 15 min. Following this sections were washed in PBS, incubated in HCl, then incubated in Borax, washed again in PBS then blocked in normal horse serum before incubating with the primary BrdU antibody (1:200) overnight at 4^°^C. On day 2 the sections were washed in PBS, incubated with the secondary antibody (anti-mouse, 1:200) for 30 min, washed in PBS, incubated in ABC reagent (Vector ABC Elite kit mouse) for 30 min, washed in PBS then SG substrate added until sufficient colour developed (2-10 min). Sections were then mounted on silanized slides. The total number of BrdU positive cells in the dentate gyrus were counted following stereological rules. Every 6^th ^section was selected and 8 sections were stained for each animal. The average number per section was calculated. There were 6 animals in each treatment group. Therefore, the overall average per group was then calculated. The values are the average number of cells per animal. The doublecortin stained neurons were counted by the same method so they are also expressed as the average number per animal.

BrdU is a pyrimidine analogue of thymidine, it is selectively incorporated into the DNA of cells during the S phase of the cell cycle, therefore, it is an indicator of proliferating cells.

#### Double Cortin (DCX) immunohistological analysis

Excess cryoprotection solution was removed, sections washed in PBS, incubated in H2O2, washed in PBS, blocked in rabbit serum (5%) for 1 h then incubated in primary antibody (goat anti-DCX, 1:200) overnight at 4 C. The following day the sections were washed in PBS, incubated in secondary antibody (Vecta ABC kit anti-goat, 1:400) for 90 min, washed in PBS, incubated in ABC reagent for 90 min, washed in PBS, incubated in SG substrate until sufficient colour developed, then finally washed in diH2O before being mounted on salinized slides. DCX is a microtubule associated protein expressed almost exclusively in immature neurons. The number of new neurons observed on each section were counted and at least 8 sections were analysed per animal.

### Statistical analysis

Results were analysed using Prism Graphpad software. Values are expressed as the mean ± SEM. Data was assessed using Students t-test and values were considered statistically significant if *p *< 0.05.

## Competing interests

Prof. Holscher is a named inventor on a patent application of liraglutide as a treatment of neurodegenerative disease, and on a second patent application of lixisenatide as a treatment of neurodegenerative disease.

## Authors' contributions

KH conducted the experiments, CH wrote the paper and raised the research funds. All authors read and approved the final manuscript.

## Authors' information

Dr. Kerry Hunter is a post-doc in the lab of Prof. Christian Holscher. Prof. Holscher is the group leader of the Neuroscience research group at the University of Ulster. He has a long track record in investigating the effects of incretin hormones and analogues on neurodegeneration.
